# Prevalence of Gastrointestinal Parasites in Wild Asian Elephants (*Elephas maximus*) at a National Park in Eastern Thailand

**DOI:** 10.3390/biology15040313

**Published:** 2026-02-11

**Authors:** Supakarn Kaewchot, Suporn Thongyuan, Supaphen Sripiboon, Rattanawat Chaiyarat, Pithak Yingyong, Watanyu Bunsermyos, Thitichai Jarudecha, Pornchai Sanyathitiseree

**Affiliations:** 1 The Graduate School, Faculty of Veterinary Medicine, Kasetsart University, Bangkok 10900, Thailand; supakarn.kae@ku.th; 2 Department of Veterinary Public Health, Faculty of Veterinary Medicine, Kasetsart University, Nakhon Pathom 73140, Thailand; fvetspty@ku.ac.th; 3 Department of Large Animal and Wildlife Clinical Sciences, Faculty of Veterinary Medicine, Kasetsart University, Nakhon Pathom 73140, Thailand; supaphen.s@ku.th; 4 Faculty of Environment and Resource Studies, Mahidol University, Nakhon Pathom 73170, Thailand; rattanawat.cha@mahidol.ac.th; 5 Angkepnam Bang Phra Non-Hunting Area, Protected Area Regional Office 2 (Sriracha), Department of National Parks, Wildlife and Plant Conservation, Chonburi 20110, Thailand; pithak.yy34@gmail.com; 6 Faculty of Agriculture, Kasetsart University, Bangkok 10900, Thailand; watanyu.bun@ku.th; 7 Department of Veterinary Nursing, Faculty of Veterinary Technology, Kasetsart University, Bangkok 10900, Thailand; thitichai.j@ku.th

**Keywords:** helminth, prevalence, wild Asian elephants, mixed infections, habitat connectivity

## Abstract

Protected national parks continue to face increased pressure from the expansion of human–wildlife interface zones, where habitat encroachment promotes human–wildlife contact and zoonotic disease transmission. Gastrointestinal parasites (GIPs) are a significant health issue in wild Asian elephants (*Elephas maximus*), affecting their fitness, survival, and potential for cross-species transmission. This study aimed to investigate the prevalence of GIPs among wild elephants at Khao Sip Ha Chan National Park in eastern Thailand. Direct smear, formalin-ethyl acetate sedimentation, flotation, and McMaster egg per gram (EPG) counting were used to examine 135 fecal samples from three populations. The findings showed that nematodes (Strongyle-type and *Strongyloides* spp.) and trematodes (*Paramphistomum* spp. and *Fascioloides jacksoni*) were identified. All samples were positive for at least one parasite species, and 84.4% were affected by mixed infections. The prevalence of Strongyle-type and *Strongyloides* spp. varied significantly among the studied populations, but *Paramphistomum* spp. had a moderate prevalence, and *Fascioloides jacksoni* had a low prevalence. The high parasite burden could be explained by environmental factors, host density, and movement patterns influencing parasite transmission, and these factors should be further investigated. These findings provide crucial baseline data and underscore the need for integrated parasite surveillance, alongside long-term conservation and future research.

## Simple Summary

Wild Asian elephants (*Elephas maximus*) living near agricultural lands often come into contact with livestock, increasing the risk of cross-species disease transmission. This study investigated gastrointestinal parasites in wild elephants at Khao Sip Ha Chan National Park, eastern Thailand, an important area within the Eastern Forest Complex where human–elephant interactions are frequent. Fecal samples (*n* = 135) were collected from three wild elephant populations and examined using four standard parasitological methods—all samples contained at least one parasite species, with a high rate of mixed infections. The most common parasites were strongyle-type and *Strongyloides* spp. nematodes, followed by the trematodes *Paramphistomum* spp. and *Fascioloides jacksoni*. Parasite prevalence and intensity varied between populations, likely influenced by environmental factors and proximity to livestock. These results highlight the importance of long-term parasite monitoring in wild elephants and support a One Health approach to managing wildlife–livestock interfaces for conservation and disease prevention.

## 1. Introduction

The Eastern Forest Complex (EFCOM) represents one of Thailand’s most ecologically significant conservation networks, comprising a mosaic of interconnected protected areas across seven eastern provinces. Ranging in elevation from 80 to 1600 m above sea level, the EFCOM supports diverse forest types—including moist evergreen, dry evergreen, and mixed deciduous forests—and plays a critical role in sustaining Thailand’s biodiversity. The complex covers more than 2240 km^2^ and harbors key wildlife species, including the endangered wild Asian elephant (*Elephas maximus*, Cuvier 1978), gaur (*Bos gaurus*, Smith 1827), banteng (*Bos javanicus*, d’Alton 1823), sambar deer (*Rusa unicolor*, Kerr 1972), and various mesocarnivores and avian species. According to the 2021 population survey conducted by the Chachoengsao Wildlife Research Station, the EFCOM supports approximately 463 wild Asian elephants, highlighting its critical role as one of Thailand’s key strongholds for wild elephant conservation.

Within this ecological network, Khao Sip Ha Chan National Park serves as a critical foraging area and movement corridor for wild elephants. Its proximity to agricultural lands and livestock areas creates dynamic interfaces between wild and domestic animals. Such interface zones facilitate wildlife movement but simultaneously increase opportunities for cross-species transmission of pathogens, including gastrointestinal parasites (GIPs). The park, recognized as one of Thailand’s most crucial wild elephant habitats, remains vulnerable to anthropogenic pressures, including agricultural expansion, encroachment, and livestock grazing. Human-driven landscape modification and resource sharing—such as waterholes, salt licks, and crop fields—have enhanced habitat connectivity and the potential for pathogen transmission across wildlife–livestock–human interfaces [1].

Despite the ecological and cultural significance of wild Asian elephants, data on GIP infection in free-ranging populations remain limited. Most available studies have focused on captive elephants, offering valuable insights into parasite diversity and associated health impacts [2,3,4]. For example, captive elephants in Nepal exhibited GIP prevalence rates of up to 95.2% across 17 parasite species [5], while a study in Chitwan National Park reported a prevalence of 47.6%, dominated by nematodes [6]. In Thailand, molecular detection of nematodes, including *Strongyloides*, *Oesophagostomum dentatum* (Rudolphi, 1803), and *Ancylostoma* spp. (Dubini, 1843), was reported in wild elephants at the Salak Phra Wildlife Sanctuary in western Thailand [7]. However, the epidemiology of GIPs in wild populations remains poorly characterized, despite their potential to influence health, reproduction, and population viability. Understanding the status of parasite infections in wild elephants is critical, as it informs conservation health strategies, aids early detection of emerging zoonotic threats, and supports One Health approaches to mitigate disease risks at the wildlife–livestock–human interface [1,8].

Wild and captive Asian elephants are highly susceptible to GIPs in their natural habitats and under human care [3]. These infections can cause weight loss, reduced fitness, and deterioration in overall health, ultimately threatening population viability [4]. Documented GIPs in elephants include trematodes such as liver flukes (*Fasciola* spp., Linnaeus 1758) [4,6], and *Fascioloides jacksoni* (syn. *Fasciola jacksoni*, Cobbold 1869) [9], cestodes such as tapeworms (*Anoplocephala* spp., Blanchard 1848) [10,11], and numerous strongyle-type nematodes, including Cyathostominae (Strongylidae), the genera *Murshidia* (Cobbold, 1882)*, Quilonia* (Lane, 1914), *Bathmostomum* (Railliet & Henry, 1909) and *Equinurbia* (Lane, 1914)*,* Ancylostomatidae (Dubini, 1843) (hookworms), especially *Grammocephalus* spp. (Railliet & Henry, 1910) [6,10,11,12,13,14]. Among these, nematodes are the most frequently recorded, with heavy infections causing tissue damage, impaired health, and increased morbidity and mortality [15]. In captive settings, such as Kerala, India, gastrointestinal nematode infections have been associated with recurrent clinical illness, including colic, diarrhea, and dependent edema [3,16,17]. In Myanmar’s timber elephants, roundworms and liver flukes were identified as direct causes of 8% of recorded deaths and as contributing factors in an additional 13% of fatalities due to generalized weakness [18]. Beyond elephants, cross-host GIP infections have been widely documented. In interface habitats shared by wild ungulates, livestock, and non-human primates, six zoonotic gastrointestinal helminths—*Trichuris* spp. (Roederer, 1761), *Trichostrongylus* spp. (Looss, 1905), *Oesophagostomum* spp. (Molin, 1861), and *Strongyloides*—were detected across multiple host species [19]. Their results show that livestock may harbor greater parasite richness than sympatric wild ruminants, thereby increasing the risk of spillovers. Similarly, in regions with high livestock presence, anthropogenic land-use changes have been shown to increase parasite diversity in wildlife, likely through spillovers from domestic animals [20]. These findings demonstrate that in areas where wildlife and livestock coexist, parasite transmission between species is both likely and well documented. This provides a clear rationale for examining GIPs in wild elephants in the national park.

This study aimed to investigate the prevalence and diversity of GIPs in wild Asian elephants (*Elephas maximus*) within a national park in eastern Thailand, where there is high interaction among humans, wild, and domestic animals. Understanding parasite burdens in this ecologically vital landscape is essential for developing effective strategies for wild elephant conservation and health management. By generating baseline data on parasite infections, this research contributes to integrated One Health frameworks that link wildlife health, ecosystem stability, and community well-being.

## 2. Materials and Methods

### 2.1. Study Site and Population 

**Figure 1:**
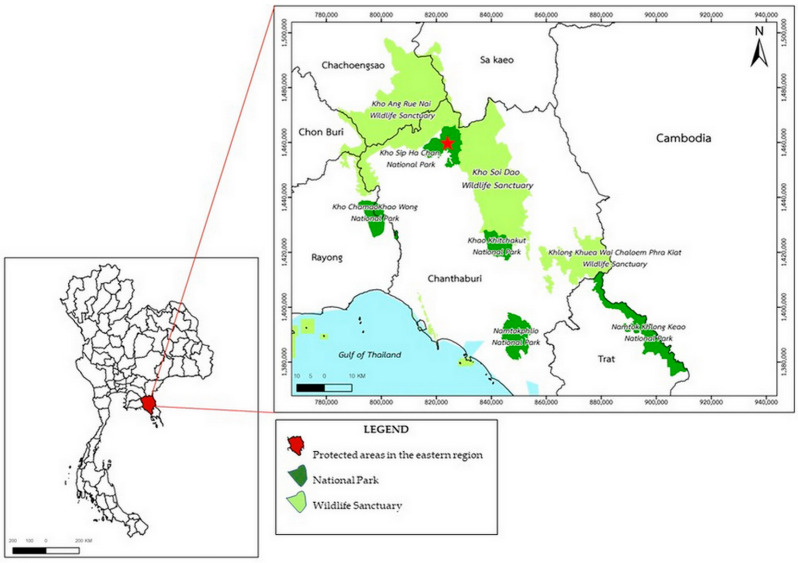
A map of Thailand highlights major protected areas across the eastern provinces, the Eastern Forest Complex (EFCOM). The red star indicates the study site at Khao Sip Ha Chan National Park, Chanthaburi Province. The map (**left**) shows the eastern region of Thailand.

Khao Sip Ha Chan National Park is located in Chanthaburi Province, eastern Thailand (approx. 12°55′23.2″ N, 101°45′2.9″ E) and spans undulating plains, lowlands, and mountain ranges, playing a crucial role in conserving wild elephants. The province spans approximately 6338 km^2^ and includes over 2075 km^2^ of conservation forest areas (Figure 1). The province features a tropical monsoon climate, with an average annual temperature of 27.4 °C and a mean rainfall of 3299.6 mm across approximately 188 rainy days. The elevations range from 100 m in valleys to peaks exceeding 900 m above sea level. This landscape supports evergreen forests, mixed deciduous forests, and grassland mosaics, providing critical habitats for elephants and other large mammals.

**Figure 2:**
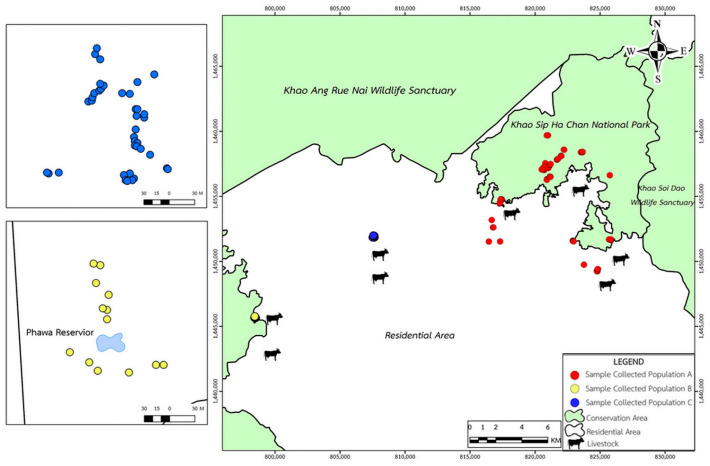
Locations of sample collection of three wild elephant populations, including population A (red dots), population B (yellow dots), and population C (blue dots).

Within this ecological matrix, Khao Sip Ha Chan National Park forms a critical conservation area of the EFCOM. The national park is ecologically connected to the Khao Soi Dao Wildlife Sanctuary in the north and the Khao Ang Rue Nai Wildlife Sanctuary in the west, forming an essential corridor for wild elephants to move and forage. For this study, sampling was concentrated in the Phawa and Khun Song Subdistricts of the Kaeng Hang Maeo District, which represent critical zones of wild elephant activity and frequent human–elephant interactions. Based on the range of areas, three populations were distinguished: Population A (60–70 individuals) inhabits the forested landscapes surrounding Khao Sip Ha Chan National Park in Khun Song Subdistrict. The terrain is dominated by evergreen and mixed-deciduous forests, with wild elephants regularly foraging along forest–agriculture edges. Population B (15–20 individuals) ranges around the Phawa Reservoir (Khlong Phawa Yai Reservoir), also in Phawa Subdistrict. This area is characterized by riparian vegetation, secondary forests, and permanent water resources that attract wild elephants year-round. Population C (20–30 individuals) occurs along the boundary with the Khao Ang Rue Nai Wildlife Sanctuary in Phawa Subdistrict. The sample collection sites for the three wild elephant populations are distributed along the interface between protected forest areas and adjacent human settlements within Khoa Sip Ha Chan National Park (Figure 2). Notably, several sampling locations are located near domestic livestock-grazing areas. This spatial overlap suggests that wild elephants and domestic livestock may utilize shared ecological resources—such as communal grazing fields, waterholes, and forest edges—thereby increasing the risk of cross-species parasite transmission. Such mixed-use zones serve as potential transmission hotspots, where helminth eggs or larvae may persist in the environment due to fecal contamination, shared water sources, or mechanical vectors. This area forms a transitional habitat between intact protected forest and agricultural land, where wild elephants frequently move across ecological and human-dominated landscapes.

### 2.2. Fecal Sample Collection

Fresh fecal samples were collected from wild Asian elephants in Khao Sip Ha Chan National Park, eastern Thailand, between June and August 2024 (rainy season), representing three populations. Wild elephants were tracked on foot, with direct observation using binoculars from a safe distance and sign-tracking based on feeding evidence [21,22]. Dung piles were located along wild elephant foraging routes by tracking signs. Only freshly deposited feces were collected to maintain high sample quality for parasitological analysis. These features included a thin mucous layer covering the dung piles, high dung moisture content, and the absence of insect activity, all of which are good indicators that a sample has been undisturbed and recently excreted [23]. Then, the researchers characterized and graded the collected samples to further confirm their freshness and consistency. All fecal samples were collected using sterile gloves to prevent contamination during handling. Freshly deposited dung piles were carefully examined, and selected material from the inner portion of each bolus was extracted to minimize contamination from soil nematodes or environmental debris. Approximately 100–200 g of feces was placed directly into labeled zip-locked bags. The geographic coordinates of each sampling location were recorded. All samples were stored immediately in a cool box and transported under chilled conditions (within 24 h) to the parasitology laboratory at the Faculty of Veterinary Technology, Kasetsart University, Bangkok. Upon arrival, samples were refrigerated at 4–6 °C and processed within 7 days, in accordance with established parasitological handling protocols [24].

### 2.3. Sample Analysis

To comprehensively detect GIPs in wild Asian elephants, four complementary laboratory techniques were employed, following standardized parasitological protocols described in Adhikari [5]. The direct smear method served as a preliminary screening tool, in which a small amount of fecal material was mixed with saline on a microscope slide and examined under light microscopy for rapid qualitative identification of helminth eggs and larvae. To enhance detection sensitivity, both flotation and formalin–ethyl acetate centrifugal sedimentation techniques were used. Flotation using a saturated salt solution allowed lighter nematode eggs to float to the surface for microscopic examination. In contrast, the sedimentation technique concentrated heavier trematode eggs by centrifugation after mixing feces with formalin and ethyl acetate. In addition to these qualitative methods, parasite burden was quantified using the McMaster egg per gram (EPG) technique, a widely used parasitological method. Fecal suspensions prepared with a flotation solution were introduced into a calibrated McMaster counting chamber. After settling, eggs were counted microscopically and multiplied by a standard conversion factor (×50) to estimate EPG values. This metric provided insights into the intensity of infection across individual wild elephants and populations.

### 2.4. Statistical Analysis

The prevalence of parasitic infections, the number of positive samples as a percentage of the total number of samples, was calculated and presented as a percentage, followed by a 95% confidence interval (CI) of infected individuals within each of the three wild elephant populations (A, B, and C). The differences in parasitic prevalence across the three distinct populations were compared using Fisher’s exact test, which is appropriate for analyzing proportions when sample sizes are small. To evaluate parasite burden, we used EPG values obtained using the McMaster technique for *Strongylus* spp. and *Strongyloides* spp. egg counts. Before selecting statistical tests, we assessed the distribution of EPG data using two steps: visual inspection via histograms and a formal test for normality. Both approaches indicated that the data did not follow a normal distribution. As a result, we applied nonparametric methods to the subsequent analyses. EPG data were described using medians and interquartile ranges (IQRs) to reflect the central tendency and spread. Differences in parasite burden across the three populations were tested using the Kruskal–Wallis test, followed by Dunn’s test to further examine pairwise differences. All statistical analyses were conducted using Stata version 17.0 (StataCorp LLC, College Station, TX, USA), with a *p*-value < 0.05 considered statistically significant.

## 3. Results

A total of 135 fecal samples of wild Asian elephants (*Elephas maximus*) were collected from inside and outside conservation areas, representing three distinct populations: Population A (*n* = 83), Population B (*n* = 13), and Population C (*n* = 39).

**Figure 3:**
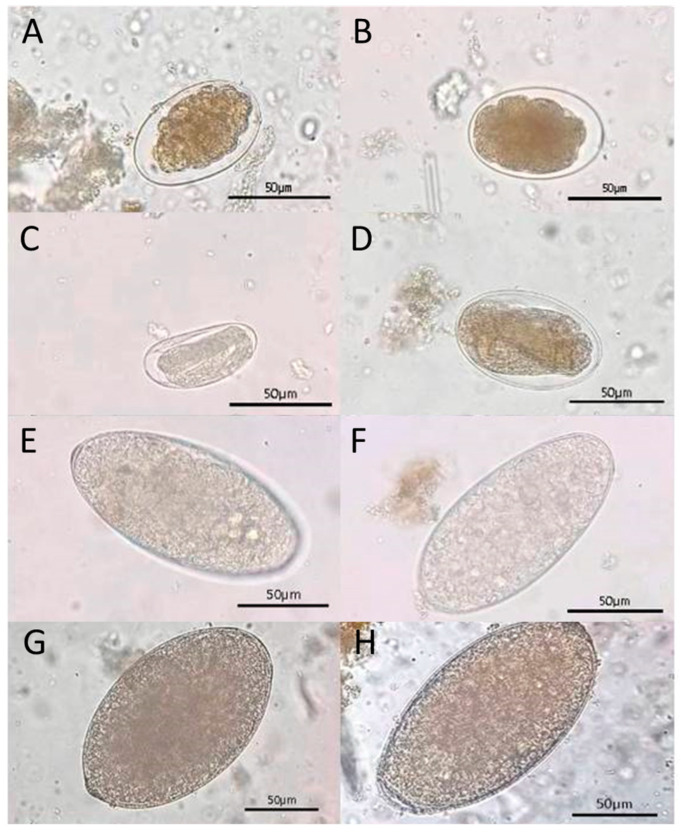
Photomicrographs of Gastrointestinal Parasites (GIPs) in fecal samples of wild Asian elephants at the national park, as follows: (**A**,**B**) Strongyle-type eggs, characterized by thin shells and coarsely granular internal material; (**C**) Embryonated strongyle-type egg, showing a developing larva within the shell; (**D**) Embryonated *Strongyloides* spp. egg, smaller in size with clearly visible larval development; (**E**,**F**) *Paramphistomum* spp. eggs, oval and translucent with loosely packed internal material; (**G**,**H**) *Fascioloides jacksoni* (eggs, showing large, oval shapes with smooth shells and granular internal contents. All images captured by light microscopy at 400× magnification. Scale bar in all panels = 50 µm.

**Figure 4:**
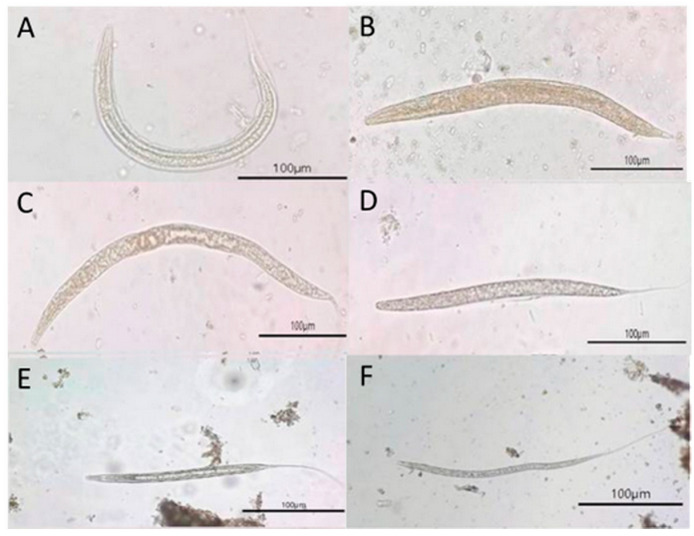
Photomicrographs of larvae and possible adult nematodes recovered from wild elephant fecal samples, visualized under light microscopy at 400× magnification, as follows: (**A**) *Strongyloides* spp. larva, displaying a characteristic coiled posture, thin translucent body, and internal features typical of rhabditiform larvae; (**B**–**F**) Non-*Strongyloides* spp. larvae showing morphological characteristics consistent with strongylid nematodes, including curved or elongated bodies, smooth cuticle, and granular or transparent internal contents. All scale bars = 100 µm.

The findings showed that all samples yielded at least one ovum or larva of GIP species, including nematodes and trematodes. The ova stage, nematode eggs, Strongyle-type and *Strongyloides* spp., and trematode eggs, *Paramphistomum* spp. and *Fascioloides jacksoni*, were identified (Figure 3). Additionally, during the larval stage, two morphologically distinct nematode larvae were identified: *Strongyloides* spp. larvae and *non*-*Strongyloides* larvae. These larval morphs represent variation in species identification and developmental stages of parasitic nematodes in wild Asian elephants (Figure 4).

### 3.1. Prevalence of GIPs

All fecal samples tested positive for at least one GIP species, with a high overall prevalence of mixed infections, 84.44% (Table 1). The prevalence of nematodes varied consistently between the populations studied (*p* < 0.05). Strongyle-type eggs were the most prevalent, at 100% in Population B and 94.9% in Population C, which are significantly higher than in Population A, 68.7% (*p* = 0.001). *Strongyloides* spp. eggs were much more frequent in Population C (87.2%) than in Population B (53.8%) and Population A (42.2%) (*p* < 0.001).

The prevalence of trematodes among the three populations did not significantly differ (*p* > 0.05). However, *Paramphistomum* spp. was present at moderate levels (46.1–67.5%) across all populations. While *Fascioloides jacksoni* eggs were detected in a few samples (5.2%), of which 6% in Population A, 15.4% in Population B, and were absent in Population C. 

There were significant differences in larval prevalence between populations (*p* < 0.001). Population C revealed the highest prevalence of *Strongyloides* spp. (87.2%) and non-*Strongyloides* spp. larva (100%).

**Figure 5:**
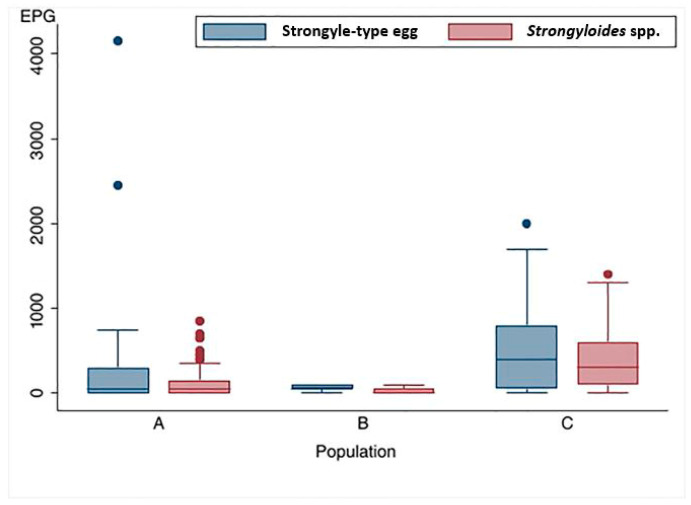
Distribution of Strongyle-type and *Strongyloides* spp. egg counts (McMaster method) among three populations of wild Asian elephants.

Distribution of Strongyle-type and *Strongyloides* spp. egg counts among the three populations were presented in Figure 5. A comparison of GIP loads and distribution across three populations revealed significant differences in Strongyle-type and *Strongyloides* spp. egg count (*p* < 0.001). The highest parasite infection intensity was observed in Population C, characterized by a broader distribution of egg counts and higher median values for both Strongyle-type eggs and *Strongyloides* spp., at 400 EPG (IQR: 50–800) and 300 EPG (IQR:100–600), respectively. The median EPG values recorded in Population C were significantly higher than those of Population A and B (*p* < 0.01).

### 3.2. Mixed Infection

**Figure 6:**
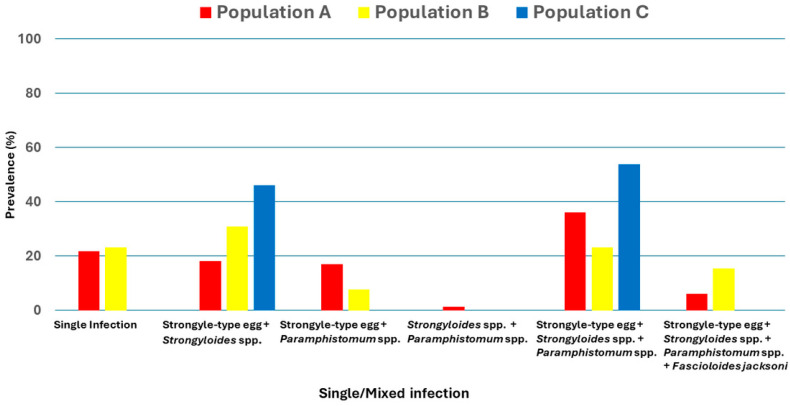
Distribution of single and mixed infection of GIPs in three populations of Wild Asian elephants.

There was a high prevalence of mixed infections across all populations (84.4%), with 100% in Population C, followed by Population A (78.3%) and Population B (76.9%). Distribution of single and mixed infections, which are combinations of two, three, and four types of GIPs among three populations were presented in Figure 6. The most common mixed infection was a combination of nematodes and trematode including Strongyle-type eggs, *Strongyloides* spp., and *Paramphistomum* spp., which were prevalent in Population C (53.8%), Population A (36.1%), and Population B (23.1%), respectively. The finding also indicated that mixed infection, a combination of Strongyle-type and *Strongyloides* spp., was significantly higher in Population C (46.1%, *p* = 0.005) than in Populations A and B (18.1% and 30.8%, respectively). However, other mixed-infection patterns showed no statistically significant differences between populations (*p* > 0.05). Interestingly, the combination of Strongyle-type and *Paramphistomum* spp. presented only in Population A (1.2%).

## 4. Discussion

In this study, we investigated the prevalence of GIPs in three wild Asian elephant populations at Khao Sip Ha Chan National Park, eastern Thailand. Four helminth types of ova stages: two nematodes (Strongyle-type and *Strongyloides* spp.) and two taxa of trematodes (*Paramphistomum* spp. and *Fascioloides jacksoni*) were identified. The results showed a high prevalence of infection across all populations studied and statistically significant differences in parasite burden and infection intensity among them. Strongyle-type and *Strongyloides* spp. were the most frequently detected. Interestingly, Strongyle-type eggs were present at high prevalence across populations, indicating that these parasites are widely distributed among wild elephant populations. Similarly, *Strongyloides* spp. were markedly more prevalent in Population C than in Population A and in Population B, highlighting substantial differences in parasite burden between the populations. These results align with previous studies conducted in India and Sri Lanka, which found that the most common nematode detected in wild elephants was *Strongylus* [11,25,26,27,28,29]. A previous study in Sri Lanka revealed a markedly higher prevalence of strongyles in wild elephants than in captive ones [29]. Additionally, a lower prevalence of strongyle type was observed among private and temple elephants compared with those managed by the forest department, likely due to differences in management practices, habitat use, and exposure to infective stages.

Several ecological and environmental factors, including habitat conditions, exposure to contaminated environments, elephant density, and movement patterns, were identified as potential drivers of gastrointestinal parasite infections. Wild elephants, due to their unrestricted movement and foraging behaviors, were more frequently exposed to infective stages of parasites in contaminated grazing areas and water sources [10,25,30]. In comparison, captive elephants were routinely dewormed and fed controlled diets, thus limiting exposure to parasitic diseases [16,29]. The significantly higher prevalence of Strongyle-type and *Strongyloides* eggs in fecal samples of wild Asian elephants in the present study suggested a more favorable environment for parasite transmission, potentially due to greater habitat fragmentation, higher humidity, and increased contact with infected dung and water sources. In addition, studies in Myanmar and Malaysia have shown that changes in nematode presence are significantly influenced by environmental determinants, seasonality, and host immunity [17,31], further supporting that the dynamics of these factors shape infection dynamics in this study. Differences in parasite burden between wild and captive elephant populations further emphasize the role of management strategies in controlling parasitic infections. It has been reported that wild elephants tend to have much higher strongyle infection rates than captive and semi-captive elephants, attributed to greater exposure to the environment, free-range foraging, and limited medical intervention [10,32]. Some captive elephants carry GIPs despite these protective measures, but have lower parasite loads than wild elephants [10].

In the present study, trematodes (*Paramphistomum* spp. and *Fascioloides jacksoni*) were detected, with moderate prevalence estimates of 46.1–67.5% for *Paramphistomum* spp. and a low prevalence rate for *Fascioloides jacksoni* (0–15.4%). The lack of a significant difference among the three populations suggested a relatively even distribution across the study area. The absence of *Fascioloides jacksoni* in Population C is noteworthy, as previous studies have reported considerable variation in trematode prevalence among elephant populations. For instance, a high prevalence of *Fasciola* spp. infection was reported in wild elephants in India [4] and Sumatran elephants [17,33]. In Nepal, both captive and wild elephants were found to be infected with *Fasciola* spp., while in Sri Lanka, high prevalence of *Fascioloides jacksoni* was reported, indicating the widespread distribution of Fasciola spp. and *Fascioloides jacksoni* among Asian elephants [6,9,34,35]. Given these previous findings, the low prevalence of *Fascioloides jacksoni* observed in this study might reflect regional differences in parasite transmission dynamics. The relatively low prevalence of *Fascioloides jacksoni* observed in this study might be attributed to differences in environmental conditions, host exposure, and the availability of intermediate snail hosts, which are essential for the parasite’s life cycle. *Fascioloides jacksoni* requires specific freshwater snails as intermediate hosts to complete their development [36]. The absence of *Fascioloides jacksoni* in Population C indicates that these wild elephants have limited access to water sources that support snail populations or forage in ways that reduce their risk of infection. Additionally, the movement patterns and habitat use of wild elephants may further influence infection rates, with populations with greater access to diverse landscapes potentially experiencing lower parasite burdens.

Rumen flukes (*Paramphistomum* spp.) are common GIPs of large herbivores, including elephants. These trematodes primarily inhabit the forestomach compartments, where they can cause subclinical infections or severe gastrointestinal disturbances [37]. The moderate prevalence reported in this study is consistent with previous studies conducted in Sri Lanka, where *Paramphistomum* spp. infection has commonly been reported in wild elephants [4,10]. In Thailand, previous studies have reported a high prevalence of *Paramphistomum* spp. infection rates in cattle with a detected prevalence above 90% [38,39]. The infection of *Paramphistomum* spp. in wild elephants indicates a common route of parasite transmission between domestic and wild herbivores, which could be further promoted by shared grazing pastures and water sources.

Furthermore, several studies have reported mixed gastrointestinal parasitic infections in wild Asian elephants across their habitat distribution [4,17,31]. This study found a remarkably high prevalence of mixed infections among wild elephant populations. The presence and mixed infections among multiple helminth species in these wild elephant populations are consistent with reported research in Sri Lanka, where mixed infections were found at 47.1%, significantly greater than single infection (21.2%) [9,10]. Similarly, research conducted in South Wayanad, India, showed that multiple parasitic species were significantly more abundant in continuous habitats, likely due to higher biodiversity and greater freedom of movement among elephant populations, which facilitates parasite transmission [11,17]. Increased exposure to diverse grazing areas and contaminated environments has been suggested as a key factor contributing to mixed infections in wild elephants. In this study area, wild elephants frequently venture outside conservation forest boundaries into agricultural lands and human settlements, where they share grazing areas and water sources with livestock such as cattle and buffalo. This overlapping habitat could further promote parasite transmission between species, increasing the likelihood of mixed infections.

The most common mixed infection detected in this study was Strongyle-type eggs, *Strongyloides* spp., and *Paramphistomum* spp. The higher parasite burden in Population C suggested that environmental factors, including humidity, soil conditions, and host density, may facilitate parasite transmission. Similar to findings from studies conducted in Sri Lanka and India, wild elephants were more likely to have multiple parasitic infections than their captive counterparts, possibly due to greater exposure to infective parasite stages in their natural habitats [2,25].

Mixed infections of Strongyle-type eggs and *Strongyloides* spp. have been widely reported in wild elephants [26,40]. The frequent co-occurrence of these nematodes suggests shared transmission routes, likely through contaminated grazing areas, water sources, or soil. Many studies reported the occurrence of *strongyles* and *Strongyloides* spp. infections in areas where wild elephants can access shaded, humid environments conducive to larval maturation [31]. Moreover, elephants with high parasite loads often experience reduced immunity, making them more susceptible to additional infections [41]. This pattern has been recorded in both wild and semi-captive elephants, wherein mixed infections observed in elephants traversing fragmented landscapes were more severe than those observed in elephants in continuous habitats [11,17].

This study highlights the high prevalence and diversity of GIPs among wild Asian elephants. The detection of both nematode and trematode species, particularly *Strongylus* spp. eggs and *Strongyloides* spp., which are the most dominant—indicates that wild elephants are consistently exposed to infective stages in their natural environment. Notably, the high rate of mixed infections in Population C suggests that ecological factors such as humidity, habitat fragmentation, host density, and proximity to domestic livestock significantly influence parasite transmission dynamics. The survey area encompasses buffer zones, communal grazing fields, water sources, and wetlands frequently used by both wild elephants and domestic livestock, such as cattle and buffalo. This landscape overlap raises concerns about cross-species parasite transmission, particularly for shared helminths such as *Paramphistomum* spp. and *Fascioloides jacksoni.* Although the latter was found at low prevalence, likely due to ecological constraints (e.g., limited availability of aquatic intermediate hosts), its zoonotic potential remains important.

The presence of ruminants near forest edges may serve as reservoirs, contaminating soil and water bodies with parasite eggs and larvae, thereby increasing the risk of infection for wildlife. These findings align with global research indicating that parasites with multi-host capacity can spill over across species in shared habitats [42,43]. For example, cross-infections among trematodes at the livestock-wildlife interface have been reported in Uganda [44] and Sri Lanka [9]. In Thailand, similar spillover risks were observed in the Huai Kha Khaeng Wildlife Sanctuary, a World Heritage Site located in western Thailand along the border with Myanmar, where both wild banteng and domestic cattle harbored strongyle-type and *Fasciola* spp. eggs [45]. Environmental conditions, such as rainfall, humidity, and soil moisture, further amplify the risk of parasite transmission. These factors have been shown to enhance parasite survival and development in multi-host systems [46]. Despite formal restrictions, informal livestock grazing within forest buffer zones remains common in Chanthaburi Province, potentially facilitating the transmission of parasitic diseases. The primary limitation of this study is the use of snapshot sampling. Data collection confined to a single rainy season may not accurately represent annual parasite dynamics, as interannual climate fluctuations—such as variations in humidity and temperatures significantly alter transmission risks and larval development rates. Future studies should implement multi-year longitudinal samplings to capture the impact of interannual climate variability on parasite prevalence. Morphological identification via fecal analysis provided limited species-level resolution, particularly for Strongyle-type nematodes. The high degree of morphological similarity between eggs and larvae in fecal samples often masks true biodiversity. Consequently, parasite diversity may be underestimated. Future studies should incorporate molecular tools, such as PCR or DNA metabarcoding, to enhance detection sensitivity and bridge these diagnostic gaps. The small sample size, particularly within Population B, serves as a constraint when investigating nuanced ecological effects, such as the impact of parasitism on population structure. While these sizes are often unavoidable in wildlife research, they limit the generalizability of the findings. To mitigate this in future research, “more robust statistical frameworks—including species abundance curves or targeted sampling of high-intensity hosts—could be employed to maximize the inferential value of limited data. Finally, this study infers cross-species transmission based on the presence of typical livestock-associated GIPs in elephants, coupled with spatial overlap. However, without genetic confirmation (e.g., DNA sequencing of identical parasite strains), the correlation remains. Utilizing genetic methods is essential to definitively confirm the direction and frequency of transmission between species in shared habitats. Given the evidence of spatial overlap, future research should move beyond presence or absence data to investigate the mechanism of transmission, which could include environmental sampling of shared water sources and grazing patches to detect larval persistence in the soil, and social network analysis of elephant herds to see how movement patterns correlate with parasite intensity and transmission clusters.

In this study, the presence of zoonotic parasites, such as *Strongyloides* spp. and *Fascioloides jacksoni*, has implications that extend beyond conservation, affecting One Health concerns for both humans and livestock. We recommend implementing longitudinal, molecular-based surveillance to identify parasite species and their sources of infection. Spatial modeling of transmission hotspots and land-use planning that limits shared access to critical habitats will be essential. Collaborative efforts between conservationists, veterinarians, public health agencies, and local communities are urgently needed to mitigate parasitic risks in human-elephant interface zones.

## 5. Conclusions

Our study is the first full-scale investigation of the prevalence of GIPs in wild Asian elephants (*Elephas maximus*) in the study area, Khao Sip Ha Chan National Park, eastern Thailand, using fecal examination methods such as direct smear, flotation, sedimentation, and the McMaster technique. Our results showed a high and consistent burden of helminth infection, with strongyle-type eggs and *Strongyloides* spp. predominant, which varied significantly among the distinct populations of wild elephants. These findings underscore the importance of including parasitological surveillance in conservation interventions for wild elephants at high risk of cross-species transmission, particularly in human-elephant interface zones. Finally, the result demonstrates the importance of a One Health strategy that integrates wildlife, livestock, and human communities in addressing parasite-induced health and conservation issues.

## Figures and Tables

**Table 1:** Prevalence of Gastrointestinal Parasites (GIPs) infection in three distinct wild Asian elephant populations.

GIPs			Prevalence of GIPs Infection			Fisher’s Exact Test	*p*-Value
		Total (*N* = 135) % (95% CI)	Population A (*n* = 83) % (95% CI)	Population B (*n* = 13) % (95% CI)	Population C (*n* = 39) % (95% CI)		
**Egg**	**Nematode**						
	Strongyle-type	79.3 (72.4–86.1)	68.7 (57.5–78.4)	100 (75.3–100)	94.9 (82.7–99.4)	14.84	0.001
	*Strongyloides* spp.	56.3 (47.9–64.7)	42.2 (31.4–53.5)	53.8 (25.1–80.8)	87.2 (72.6–95.7)	21.88	<0.001
	**Trematode**						
	*Paramphistomum* spp.	61.5 (53.3–69.7)	67.5 (56.3–77.3)	46.1 (19.2–74.9)	53.8 (37.2–70.0)	3.51	0.173
	*Fascioloides jacksoni*	5.2 (1.4–8.9)	6.0 (2.0–13.5)	15.4 (1.9–45.4)	0 (0–9.0)	5.0	0.082
**Larval Stage**							
	*Strongyloides* spp.	55.6 (47.2–63.9)	39.8 (29.2–51.1)	61.5 (31.6–86.1)	87.2 (72.6–95.7)	24.37	<0.001
	Non-*Strongyloides* spp.	83.0 (76.6–89.3)	72.3 (61.2–81.5)	100 (75.3–100)	100 (91.0–100)	17.37	<0.001
**Mixed infection**		84.4 (78.3–90.6)	78.3 (67.9–86.6)	76.9 (46.2–95)	100 (91.0–100)	10.12	0.006

## Data Availability

The data presented in this study are available from the corresponding author upon request.

## Abbreviations

The following abbreviations are used in this manuscript:

GIPs	Gastrointestinal parasites
EFCOM	The Eastern Forest Complex
EPG	Eggs Per Gram
PCR	Polymerase chain reaction
DNA	Deoxyribonucleic acid
